# Vaccination with Animal and Human Virus

**Published:** 1882-01

**Authors:** 


### VACCINATION WITH ANIMAL AND HUMAN VIRUS.

Dr. E. R. Warlomont, of Brussels, in a lengthy paper contributed to the *British Medical Journal*, discusses very fully the relations of animal vaccination to vaccination and re-vaccination in general. In view of the general prevalence of small-pox throughout the United States at present, we give an abstract of the article :

It has been asked, If human vaccine be a protection against small-pox, why, where there is no want of it, should animal vaccine be used ? The answer is, That this substitution is necessary to satisfy certain doubts, fears, prepossessions, and, perhaps, prejudices. In countries where vaccination is legally compulsory, the duty of the State is to put into the hands of the public a vaccine lymph which shall be beyond any suspicion of diathetic adulteration. This guarantee cannot be given with humanized vaccine, as proved by the existence of vaccinal syphilis ; whilst, by the use of calf-lymph, a careful operator is always certain to offer such a guarantee to the persons inoculated by him. It is not sufficient that vaccine obtained from calves should be innocuous ; it is also necessary that it should be inoculable, and that it should preserve us from variola.

To the question whether vaccination by calf-lymph protects from small-pox like humanized vaccine, the answer is, that, up to the present time, no one has contested the point. Out of more than ten thousand children vaccinated at Brussels, with animal vaccine, from 1869 to 1870, not one case was known to have been attacked by the epidemic of 1870 and 1871. The same immunity was shared by revaccinated cases, which, though not nearly so numerous, were, at the same time, in the foci of the epidemic.

Finally, what is the degree of susceptibility of the human species to animal vaccine ? The answer may unhesitatingly be given, that it is equal, if not superior, to that for humanized vaccine. When the calf-lymph is inoculated direct, taken from good pustules of the proper age, no other failures are known but those resulting from the manipulation of the operator. Out of three hundred children thus vaccinated since May 1st of this year (1881) not one puncture has failed to produce good pustules.

With regard to preserved vaccine, the following statistics are given : In 1870 and 1871, thirty-three of the most reliable physicians in Belgium reported the results obtained by them in vaccinations and revaccinations, by means of calf-lymph on points sent out by the State Vaccine Institute of Brussels. These results are: 1. In vaccinations, out of a total of 500 cases, there were 479 successful, being at the rate of 96 per cent. 2. In revaccinations, out of a total of 5,448 cases, there were 3,419 successes, being at the rate of 62 per cent.

These figures are very much larger than any brought out elsewhere, for, in England, the best vaccinators only reckon their successful cases at the rate of 90 per cent. after the use of humanized vaccine preserved in tubes, and at 95 per cent. when ivory points bearing the same kind of vaccine are employed.

This fundamental principle, based upon prophylaxis, is generally accepted. Everyone knows that prevention is better than cure, and everyone wishes to take care of number one. With regard to small-pox, the prophylactic power of vaccine is universally recognized; and, if it could for one moment be forgotten, do not the declamations of the antivaccinationists incessantly stimulate proper conviction, through the reaction which ever follows their efforts? Everyone wishes to be protected from small-pox, and every one knows how to do so. Hence the eagerness of all classes of the public for vaccination and revaccination, an eagerness actually fostered by the contradictions of its efficacy brought forward by the League, which only keeps the subject of vaccination before the attention of the public.

A certain restraint, however, checks this spontaneous and salutary movement; where animal vaccination is unknown the spectre of vaccinal syphilis is present. In wishing to protect themselves from one evil, some persons fear to expose themselves to another. The possession of animal vaccine removes this restraint. We know that henceforth we may be revaccinated without having anything to fear. Hence large requirements which the production of humanized vaccine cannot meet, for the demand exceeds the supply. Confidence in calf-lymph is fully justified. Of syphilis, there is, of course, no question. There still remains tuberculosis, which, it has lately been suggested, might be communicated to a patient through animal vaccine. For this purpose, theoretical arguments, already confuted, have been relied on. It must, however, be acknowledged that these arguments have assumed an inestimably more serious phase since tuberculosis has been shown to be entitled to rank amongst the microbic infectious diseases, so that this hypothesis, which does not lack support, can at least no longer be rejected from amongst the number of possible occurrences. It is the more serious, inasmuch as cow-pox itself does not escape from this accusation, since bovine animals are subject to a disease which, though not precisely tuberculosis, is certainly akin to it.

The bovine race suffer from the pommelere, or pearl-disease, which cannot be considered as true tuberculosis. But it must be noted that the transmission of tuberculosis—excluding an immense majority of these cases which are untrustworthy—is obtained by the inoculation of the tuberculous matter itself. No one has ever anywhere spoken of transmission obtained through the serum of a tuberculous animal. We must therefore place our reliance on the inoculability of constitutional tuberculosis. It is affirmed that there does not exist any fact or proof whatsoever in support of a transmission of this nature.

A great movement of opinion has lately arisen in England and Germany against the use of milk obtained from cows suffering from tuberculosis or the pearl-disease. The great mortality amongst children brought up on the bottle was attributed to a great extent to the use of this milk. The accusations became more and more pressing and precise. Berlin was agitated by them, and a commission was nominated in that city to collect the statistical materials necessary for the solution of the question. The conclusions to which the commission came, formulated at the end of last year by Prof. Virchow, one of its members, have been negative, in the sense that rigorous examination of the facts have not given warrant for the affirmation of transmission of bovine tuberculosis to man by milk. There only then remains pure hypothesis, which can be easily constructed in relation to every kind of medication, alimentation, cohabitation—in fact of every habit.

Thus, it may be affirmed that neither syphilis, nor tuberculosis, nor glanders, nor anthrax, nor any diathetic disease, can be inoculated into the human race by animal vaccine. This, however, is dependent upon one condition, which is : that the vaccine, whether living or preserved, should be taken from a healthy animal, and collected at the required moment ; this is the A B C of the whole question. If preserved, it should be done according to methods which place it beyond the possibility of any subsequent change. For this purpose there are two methods of preservation which perfectly attain this end :

that of dried vaccine on ivory points, and that of liquid defibrinated vaccine introduced into tubes.

If, then, the public has recourse to vaccination and revaccination, it obeys a sentiment of confidence which is universally justified, and which is likely to spread in proportion as animal vaccination is more known. There is still another circumstance which will contribute to justify the demand for revaccination. The public as well as the profession now know without possibility of doubt that the immunity conferred by vaccine wears out with time, and that we must consequently resort to periodical revaccination. On this point, the following formula is laid down: Be revaccinated, how frequent soever may have been the preceding revaccinations, whenever there is an epidemic of small-pox; and the more quickly in proportion that the epidemic is serious. This principle is destined, if it be accepted—and it certainly will be accepted by prudent persons—to increase tenfold in the course of time the demand for vaccine matter; and consequently, in a season of epidemic, the production of human vaccine will always be smaller than this demand.

When a child is brought back at the expiration of the first seven days, if it be revaccinated on the spot, even with its own vaccine lymph, it may be that there will be a fresh eruption, feeble for the most part, but occasionally showing all the signs of classic vaccinal pustule. What conclusion is to be drawn, if not that the first inoculation, insufficient to protect the subject against a second vaccinal impregnation, was *a fortiori* insufficient to guard it against variola? Hence the necessity of fresh insertions until the complete exhaustion of vaccinal receptivity. This is termed *vaccinisation.* Thus every child brought back at the end of eight days should be revaccinated on the spot, even with its own vaccine, if it be in proper condition. If this second vaccination answer well, a third should be performed, and so on till the patient be completely *vaccinised.*

Such are the fresh considerations we have to weigh in favor of animal vaccination. It has been objected that Jenner's opinion was against it; but this argument surely has no weight. In matters of experimental science, the predictions of the greatest geniuses only show the imprudence of those who express them. Facts have decided against the predictions expressed on this subject by the immortal discoverer of vaccination.

Animal vaccine has been used in Belgium for about sixteen years.

The Government has bestowed its patronage on that material since 1868, propagates it by the labors of its agents, and at the present time is studying how to still further place it free of charge in the hands of all. When the Government has achieved this result, it will examine the question of compulsory vaccination. The method of proceeding in England has not been so wise ; here they have put the cart before the horse.

The following conclusions are submitted :

1. In all countries, the placing of animal vaccine in the hands of the public removes the only obstacle which still keeps recalcitrants from the lancet of the vaccinator.

2. In countries where vaccination is compulsory, it is necessary that the Administration should place within the reach of families vaccine lymph which calms their fears concerning the transmission of diathetic affections.

3. Animal vaccine is, then, the necessary succedaneum for human vaccine. To propagate it to such an extent that it shall never fail the requirements of revaccination in a season of epidemic, however extensive they may be, is the first and most imperious duty of Governments in the domain of State medicine.

